# Advanced Materials, Structures, and Technologies for Thin-Film Light-Emitting Diodes

**DOI:** 10.3390/ma19051012

**Published:** 2026-03-06

**Authors:** Carmela Tania Prontera, Marco Pugliese

**Affiliations:** 1 Laboratory of Hydrogen and New Energy Vectors (TERIN-DEC-H2V) ENEA–C.R Brindisi, S.S.7 Appia, km 706, 72100 Brindisi, Italy; 2 CNR-NANOTEC—Institute of Nanotechnology, c/o Campus Ecotekne, Via Monteroni, 73100 Lecce, Italy

## 1. Introduction

Recently, thin-film light-emitting devices have attracted considerable attention due to their potential application in displays and lighting. Their advantages include compatibility with flexible substrates and solution-based deposition techniques, as well as reduced manufacturing costs thanks to minimal material usage in thin film form [1,2,3]. However, numerous scientific and technological challenges still need to be overcome before they can achieve full technological maturity. The different topics addressed in this field include new material design, device engineering, processing and advanced characterisation techniques. This Special Issue presents some recent contributions on this topic and the main results are briefly discussed below.

## 2. An Overview of Published Articles

### 2.1. Innovative Materials

In the development and study of new materials for thin-film light-emitting devices, it is useful to distinguish between inorganic and organic materials.

In the case of inorganic materials, the development of efficient and stable phosphors is essential for enhancing the performance of white light-emitting diodes (LEDs), particularly with regard to colour rendering and the red component of the spectrum. In this context, rare-earth ion-doped materials, especially those containing Eu^3+^ ions, are of great interest as luminophores due to their well-defined electronic transitions and strong sensitivity to the crystalline environment [4]. Among the various host matrices, melilite-type structures offer high structural flexibility and a favourable crystalline field for tuning photoluminescent properties.

In this regard Li et al. (contribution 1) reported the synthesis of CaYAl_3_O_7_: Eu^3+^ phosphors for white LEDs. The resulting phosphors exhibit efficient excitation at 398 nm and the desired emission at 622 nm. Furthermore, the optimal Eu concentration was found to be x = 0.16. Eighteen experimental fluorescent spectra of Eu^3+^ ions at the Y^3+^ site were reproduced with good precision by fully diagonalising the Hamiltonian. This confirms the effectiveness of the method for studying luminescent phosphors and developing new white LEDs.

In recent decades, there has been a growing interest in using organic materials in light-emitting devices as an alternative to traditional inorganic phosphors. Organic materials can be obtained through relatively simple synthesis processes, and their optical and electronic properties can be finely tuned through appropriate molecular modifications.

Phenanthro [9,10-d]imidazole derivatives are a class of organic material that is of great interest for electroluminescent applications thanks to their structural rigidity, thermal stability and favourable electronic properties [5]. Their optical and electrochemical characteristics can be finely tuned through the targeted introduction of substituent groups, whose nature and position play a key role in determining material’s final performance. However, the influence of substituent position on physicochemical properties remains an area that requires systematic analysis.

Krawiec et al. (contribution 2) reported the synthesis of four phenanthro [9,10-d]imidazole derivatives (AM-0 to AM-3), whose thermal, electrochemical and optical properties were subsequently compared. They found that compounds with a 4-diethylaminophenyl substituent in the C2 position (AM-0 and AM-1) exhibited superior performance, showing greater thermal stability, a more favourable energy gap, and a quantum yield 15–30% higher than that of derivatives with the substituent in the N1 position. These results confirm the significant influence of functional group position on the development of high-performing phenanthro [9,10-d]imidazole derivatives for organic light-emitting diode (OLED) devices.

In addition to purely organic materials, transition metal complexes have great potential for use in light-emitting devices due to their intense emission, colour tunability, and favourable charge transport properties. Iridium (Ir^3+^) and ruthenium (Ru^2+^) complexes are particularly promising organometallic emitters for such devices. Ir^3+^ complexes exhibit highly efficient phosphorescence and tunable emission across much of the visible spectrum, while Ru^2+^ complexes are stable and electronically versatile owing to their tunable ligands [6,7]. These complexes complement and enhance the performance of traditional organic materials, helping to improve device efficiency, colour, and stability. 1H-Imidazo [4,5-f][1,10]phenanthroline derivatives are promising ligands for Ir and Ru complexes and their physicochemical properties were summarized and discussed in the review of Krawiec et al. (contribution 3), with particular focus on their application in light-emitting electrochemical cells.

### 2.2. Inkjet Printing for OLED Manufacturing

The development of OLED devices based on organic materials has stimulated growing interest in alternative fabrication techniques to conventional vacuum-based processes. In this context, printing techniques represent a promising approach to produce light-emitting devices, thanks to their compatibility with organic materials, low production costs, and the possibility of producing large-area devices on flexible substrates.

The work of Manfredi et al. (contribution 4) addresses a key challenge in the development of fully printed OLEDs by optimising the ink formulation for the inkjet deposition of electron transport layers (ETLs) without damaging the underlying layers. Integrating the printed ETL into a device yelds promising electro-optical performance (6.8 cd/A and ~8700 cd/m^2^), representing a significant step forward in the development of fully solution-processable, low-cost OLED architectures.

### 2.3. Probing OLED Degradation Through Spectroscopic Ellipsometry

The development of thin-film OLED devices requires the design of materials with suitable optoelectronic properties, as well as a thorough understanding of their structural and morphological stability within complex multilayer stacks. Interfacial interactions and thermally induced phase transitions can significantly affect the performance and lifetime of these systems. Therefore, advanced in situ characterisation techniques play a fundamental role in monitoring the evolution of thin films under accelerated operating or ageing conditions, providing key information with which to optimise materials and device architectures.

Aulika et al. (contribution 5) investigated the thermal behaviour of a multilayer structure (glass/ITO/TAPC/CBP/BPhen) in an OLED system using in situ spectroscopic ellipsometry. The identification of anomalies associated with phase transitions and interfacial rearrangements sheds new light on degradation mechanisms and may help improve the thermal stability of OLED devices.

## Data Availability

Data sharing is not applicable.
